# Upregulated anti-angiogenic miR-424-5p in type 1 diabetes (model of subclinical cardiovascular disease) correlates with endothelial progenitor cells, *CXCR1/2* and other parameters of vascular health

**DOI:** 10.1186/s13287-021-02332-7

**Published:** 2021-05-14

**Authors:** Alice Tamara, David J. Coulson, Jevi Septyani Latief, Sherin Bakhashab, Jolanta U. Weaver

**Affiliations:** 1 Translational & Clinical Research Institute, Newcastle University, Newcastle upon Tyne, NE2 4HH UK; 2 Faculty of Medicine, Universitas Indonesia, Jakarta, 10430 Indonesia; 3 Biochemistry Department, Faculty of Science, King Abdulaziz University, Jeddah, 80218 Saudi Arabia; 4 Department of Diabetes, Queen Elizabeth Hospital, Gateshead, Newcastle upon Tyne, NE9 6SH UK; 5 Vascular Biology and Medicine Theme, Newcastle University, Newcastle upon Tyne, NE2 4HH UK

**Keywords:** MiR-424-5p, IL8, CD45^dim^CD34^+^CD133^+^, *CXCR1/2*, T1DM

## Abstract

**Background:**

In spite of clinical progress, cardiovascular disease (CVD) remains the predominant cause of mortality worldwide. Overexpression studies in animals have proven miR-424-5p to have anti-angiogenic properties. As type 1 diabetes mellitus (T1DM) without CVD displays endothelial dysfunction and reduced circulating endothelial progenitor cells (cEPCs), it offers a model of subclinical CVD. Therefore, we explored miR-424-5p, cytokines and vascular health in T1DM.

**Methods:**

Twenty-nine well-controlled T1DM patients with no CVD and 20-matched controls were studied. Cytokines IL8, TNF-α, IL7, VEGF-C, cEPCs/CD45^dim^CD34^+^CD133^+^ cells and ex-vivo proangiogenic cells (PACs)/fibronectin adhesion assay (FAA) were measured. MiR-424-5p in plasma and peripheral blood mononuclear cells (PBMC) along with mRNAs in PBMC was evaluated.

**Results:**

We found an elevation of IL7 (*p* = 0.008), IL8 (*p* = 0.003), TNF-α (*p* = 0.041), VEGF-C (*p* = 0.013), upregulation of mRNA *CXCR1* (*p* = 0.009), *CXCR2* (*p* < 0.001) and reduction of cEPCs (*p* < 0.001), PACs (*p* < 0.001) and FAA (*p* = 0.017) in T1DM. MiR-424-5p was upregulated in T1DM in PBMC (*p* < 0.001). MiR-424-5p was negatively correlated with cEPCs (*p* = 0.006), PACs (*p* = 0.005) and FAA (*p* < 0.001) and positively with HbA_1c_ (*p* < 0.001), IL7 (*p* = 0.008), IL8 (*p* = 0.017), VEGF-C (*p* = 0.007), *CXCR1* (*p* = 0.02) and *CXCR2* (*p* = 0.001). ROC curve analyses showed (1) miR-424-5p to be a biomarker for T1DM (*p* < 0.001) and (2) significant upregulation of miR-424-5p, defining subclinical CVD, occurred at HbA_1c_ of 46.5 mmol/mol (*p* = 0.002).

**Conclusion:**

We validated animal research on anti-angiogenic properties of miR-424-5p in T1DM. MiR-424-5p may be a biomarker for onset of subclinical CVD at HbA_1c_ of 46.5 mmol/mol (pre-diabetes). Thus, miR-424-5p has potential use for CVD monitoring whilst anti-miR-424-5p-based therapies may be used to reduce CVD morbidity/mortality in T1DM.

## Background

Cardiovascular disease (CVD) is the leading cause of mortality worldwide, accounting for 31% of global deaths in 2016 [1]. It has been long established that the development and progression of CVD could not be separated from the presence of inflammation [2]. Although similarly regarded as an inflammatory disease, diabetes mellitus also acts as one of the most important predisposing factors for CVD. In fact, the presence of type 1 diabetes mellitus (T1DM) decreases one’s life expectancy by 13 years [3], predominantly due to cardiovascular events. Moreover, the risk of having CVD was reported to increase to 3.6 and 7.6 in men and women with T1DM, respectively [4]. We and others have demonstrated that T1DM without CVD has features of subclinical CVD [5–7]. It is associated with endothelial dysfunction and reduced indices of vascular health—circulating endothelial progenitor cells (cEPCs), Hills colonies, pro-angiogenic cells (PACs) and fibronectin adhesion assay (FAA)—known to define CVD in other patient’s groups [8, 9]. Thus, this data focuses our attention on T1DM as a model of subclinical CVD.

Recent studies discovered the differential microRNA (miR) expression in diabetic patients [10, 11]. miRNA is a small single-stranded non-coding RNA, which acts to silence the mRNA expression in post-transcriptional level [12]. Several miRNAs have been shown to play an important role in the development of CVD [12–14]. Many pro-inflammatory miRNAs have been studied, of which miR-424-5p appeared to be a marker of plaque instability [15, 16].

In vitro study of miR-424 reported a significant decrease in the proliferation of pulmonary artery endothelial cells following the overexpression of miR-424 [17]*.* Furthermore, the upregulation of plasma miR-424-5p was correlated with higher rate of vascular events such as deep vein thrombosis and vein thromboembolism [18, 19]. MiR-424-5p was also discovered to be elevated in the plasma in relatives of T1DM [20]. The uncomplicated T1DM with good diabetic control offers a model of subclinical CVD and therefore can help to understand the early mechanisms involved in development of CVD. Understanding the role of miR-424-5p in CVD enables potential use as a biomarker and therapeutic target, to reduce burden of CVD, particularly in T1DM.

Hence, we hypothesise that miR-424-5p is upregulated in uncomplicated T1DM and associated with the increased risk of CVD.

## Methods

### Study design

In this cross-sectional study, we recruited 29 T1DM patients and 20 age- and gender-matched healthy subjects. The inclusion criteria for our study were T1DM patients with good glycaemic control indicated by glycated haemoglobin level (HbA_1c_) < 69 mmol/mol or 8.5% and eGFR> 45 ml/min/1.73m^2^ with no evidence of diabetic complications, such as CVD and active proliferative retinopathy. The sample size was calculated by power calculation as previously reported [5].

T1DM patients were recruited from Queen Elizabeth Hospital Gateshead or Royal Victoria Infirmary, Newcastle, UK. A written informed consent was obtained from each subject. This study was performed in line with the principles of the Declaration of Helsinki and was approved by the NHS Health Research Authority, NRES Committee North East-Sunderland, UK (Research Ethics Committee Reference Number 12/NE/0044).

### Clinical and laboratory assessment

Peripheral blood samples were collected from all participants after an overnight fast. Plasma isolated from peripheral blood samples collected in EDTA tubes was used to assess the different levels of cytokines and angiogenic markers in the study groups. Clinical and laboratory examinations included routine laboratory tests such as full blood count, urea and electrolytes, liver function, thyroid function and HbA_1c_ and electrocardiogram, blood pressure, body weight, body height and body mass index.

### Meso Scale Discovery (MSD) assay

Plasma samples were assayed using K15050D V-PLEX Cytokine Panel 1 human kit, K15049D V-PLEX Proinflammatory Panel 1 human kit and K15190D V-PLEX Angiogenesis Panel 1 human kit (Meso Scale Discovery, Rockville, MD) in accordance with the manufacturer’s protocol. Plates were read with MSD Sector Imager 2400, and data were analysed using MSD Discovery Workbench v2.0 software.

### Flow cytometric evaluation of circulating endothelial progenitor cells

The cEPCs were defined as CD45^dim^CD34^+^CD133^+^ cells, and samples were analysed by flow cytometry on a BD FACS Canto™ II system (BD Bioscience, San Jose, CA, USA) as described previously [5].

### In vitro assays for vascular health

#### Proangiogenic cells (PACs)

Ficol density gradient protocol was used to isolate PBMCs. Fibronectin-coated 24-well culture plate was used to plate 1 × 10^6^ PBMCs in complete endothelial basal medium (EBM-2, PromoCell, Heidelberg, Germany) supplemented with 20% fetal calf serum and growth factors—human epithelial growth factor, VEGF, human basic fibroblast growth factor, recombinant human long R3 insulin like growth factor-1, platelet-derived growth factor-AA and Hep-11. Non-adherent cells were removed after 48 h. Phosphate-buffered saline was used to wash the remaining adherent cells twice and cells were further incubated for 48 h. Adherent cells were then stained on day 4 with acetylated low-density lipoprotein (Ac-LDL) and Ulex lectin (Merk, St. Louis, Missouri, US). Cells which stained positive for both Ac-LDL and Ulex lectin were classed as PACs. Fifteen random high-power fields (hpf, 200X) photographs were taken, and only dual positive cells were counted. Since PACs have been described as displaying strong neovascularization activity, this assay has been used to study neovascularization potential in studied subjects [21].

#### Fibronectin adhesion assay

PACs were detached gently from fibronectin coated 6-well culture plate using 5 mmol/L EDTA [22]. Once detached, these cells were washed in PBS and 1 × 10^5^ cells were re-plated on fibronectin coated 48-well plate in EBM-2 (PromoCell) supplemented with 5% fetal calf serum. The cells were incubated for 30 min at 37 °C as previously described [23]. Subsequently, 48-well culture plate was vigorously washed. Remaining adherent cells were stained with Ac-LDL and Ulex lectin as mentioned above. Ten random hpf (200X) were photographed and cells which were dual positive were counted.

### Expressions of miRNA-424-5p and its mRNA targets

#### Plasma samples preparation

Platelet-free plasma was obtained through consecutive centrifugations of blood samples at 500×*g* for 15 min and 13,000×*g* for another 5 min. The plasma was tested for haemolysis to ensure the samples were not contaminated with cellular miRNA. An aliquot of 200 μL per sample was transferred to a FluidX tube and 60 μl of Lysis solution BF containing 1 μg carrier-RNA per 60 μl Lysis Solution BF and RNA spike-in template mixture was added to the sample and mixed for 1 min and incubated for 7 min at room temperature, followed by addition of 20 μL Protein Precipitation solution BF. Total RNA was extracted from the samples using miRCURY RNA isolation Kit – Biofluids, high-throughput bead-based protocol v.1 (Exiqon, Vedbaek, Denmark) in an automated 96-well format. The purified total RNA was eluted in a final volume of 50 μl. Plasma miRNAs were isolated from plasma samples using RNA isolation protocol optimised for plasma by QIAGEN (Exiqon Services, Denmark). The integrity of extracted RNAs were measured using 2100 Bioanalyzer (Agilent, Santa Clara, CA, USA) with the value of 9.1–10 considered as high in integrity.

#### The sample preparation of peripheral blood mononuclear cells

Peripheral blood mononuclear cells (PBMC) were isolated from peripheral blood using Ficol (GE Healthcare, Chicago, IL, USA). MicroRNAs and mRNAs were extracted in TRIzol (Invitrogen, Carlsbad, CA, USA) and miRNEasy Kit (QIAGEN, Hilden, Germany). The integrity of the extracted PBMC RNAs were determined using 2100 Bioanalyzer (Agilent, Santa Clara, CA, USA).

#### Plasma miRNA real-time PCR

Two microliters of RNA was reverse transcribed in 10 μl reactions using the miRCURY LNATM Universal RT microRNA PCR, Polyadenylation and cDNA synthesis kit (Qiagen, Hilden, Germany). cDNA was diluted 50× and assayed in 10 μl PCR reactions according to the protocol for miRCURY LNA™ Universal RT microRNA PCR; each microRNA was assayed once by qPCR on the microRNA Ready-to-Use PCR using ExiLENT SYBR® Green master mix. Negative controls excluding template from the reverse transcription reaction was performed and profiled like the samples. The amplification was performed in a LightCycler® 480 Real-Time PCR System (Roche, Basel, Switzerland) in 384-well plates, and the amplification curves were analysed using the Roche LC software. hsa-miR-424-5p was assayed using miRCURY LNA miRNA PCR Assays with Cat number: YP00204736 (Qiagen).

Normalisation was performed based on the average of the assays detected in all samples using global mean normalisation method. For the present study, this included 7 assays hsa-miR-21-5p (TAGCTTATCAGACTGATGTTGA), hsa-miR-320a (AAAAGCTGGGTTGAGAGGGCGA), hsa-miR-23a-3p (ATCACATTGCCAGGGATTTCC), has-miR-92a-3p (TATTGCACTTGTCCCGGCCTGT), hsa-miR-223-3p (TGTCAGTTTGTCAAATACCCCA), hsa-miR-126-3p (TCGTACCGTGAGTAATAATGCG) and hsa-miR-15a-5p (TAGCAGCACATAATGGTTTGTG). All data was normalised to the average of assays detected in all samples (average – assay Cq).

#### PBMCs miRNA and mRNA real-time PCR

The 10 ng of miRNAs was reversed transcribed using the miRCURY LNA RT Kit (QIAGEN, Hilden, Germany). After 100 times dilution, cDNA underwent PCR using miRCURY LNA miRNA PCR according to the manufacturer’s protocol in a LightCycler® 480 Real-Time PCR System (Roche, Basel, Switzerland). hsa-miR-424-5p was assayed using miRCURY LNA miRNA PCR Assays with Cat number: YP00204736 (Qiagen).

All data was normalised to the average of 11 assays (hsa-miR-30e-3p, Cat number: YP00204410; hsa-miR-365a-3p, Cat number: YP00204622; hsa-miR-374b-5p, Cat number: YP00204608; hsa-miR-26b-3p, Cat number: YP00204117; hsa-miR-576-5p, Cat number: YP00206064; hsa-miR-425-3p, Cat number: YP00204038; hsa-miR-454-5p, Cat number: YP00204279; hsa-miR-769-5p, Cat number: YP00204270; hsa-miR-200c-3p, Cat number: YP00204482; hsa-miR-660-5p, Cat number: YP00205911; hsa-miR-331-3p, Cat number: YP00206046; Qiagen) detected in all samples.

Reverse transcription of 150 ng PBMC mRNAs was performed using QIAGEN RT ^2^ First Strand Kit (QIAGEN, Hilden, Germany), yielding cDNAs, which were arrayed using RT ^2^ Profiler PCR Array (QIAGEN, Hilden, Germany) containing *CXCR1* (Cat number: PPH01040F) and *CXCR2* (Cat number: PPH00608F). All Cq data was normalised to reference genes: actin-β (Cat number PPH00073G), lactate dehydrogenase A (Cat number: PPH02047H), hypoxanthine phosphoribosyltransferase 1 (Cat number: PPH01018C), ribosomal protein, large, P0 (Cat number: PPH21138F), β-2-microglobulin (Cat number: PPH01094E) and glyceraldehyde-3-phosphate dehydrogenase (Cat number: PPH00150F) yielding ΔCq. Fold change analysis was performed using 2^−ΔΔCq^ calculation.

#### Prediction model: ingenuity pathway analysis (IPA) of miR-424-5p and its mRNA targets

The functional pathway analysis of mRNAs and miRNA-mRNA relationships in relation to their implications in cardiovascular disease was conducted using ingenuity pathway analysis (IPA) software 9.0 (Ingenuity, Redwood City, CA, USA). Functional analysis using IPA offers independent target function based on (1) matching sequence of mRNA and miRNA, with weak evidence if incomplete matching, (2) correlation between the studied factors (weak evidence) and (3) direct and indirect interventional experiments (strong evidence).

The interaction site prediction between the transcripts and miR-424-5p was performed using TargetScan Human, release 7.1 (www.targetscan.org), and Diana-TarBase v8, (http://carolina.imis.athena-innovation.gr/diana_tools/web/index.php?r=tarbasev8%2Findex) [24] databases.

#### Statistical analysis

Data is presented as mean ± SD. As data was normally distributed, the difference was assessed using unpaired *t* test. Correlation between miR-424-5p expression and other markers were measured using linear regression tests. *Receiver operating characteristic* (ROC) curve analysis of miR-424-5p in study participants was carried out to assess the sensitivity of miR-424-5p as a biomarker for subclinical CVD/T1DM. Moreover, ROC curve analysis of significantly elevated miR-424-5p and HbA_1c_ was performed to determine the cutoff value for miR-424-5p upregulation. Statistical analysis was performed using SPSS 24.0 (SPSS™ Inc., Armonk, NY, USA), and the graphs were constructed using GraphPad Prism 7.0 (GraphPad software, San Diego, USA). A *p* < 0.05 was considered statistically significant.

## Results

### Characterisation of T1DM patients and healthy controls

There were 29 T1DM patients and 20 age- and gender-matched healthy controls recruited in this study. The sociodemographic characteristics and laboratory data of the studied subjects were summarised in Table 1. The mean age of T1DM group and healthy controls were 47 and 46 respectively. T1DM group had diabetes for at least 8 years and relatively good glycaemic control, mean HbA_1c_ 57.3 mmol/mol (7.4%).

**Table 1:** Clinical and metabolic characteristics of included subjects

Characteristics	Healthy control group	T1DM	*p* value
***N***	20	29	
**Age (years)**	46.5 ± 11.7	47.2 ± 12.7	0.8
**Body mass index (kg/m)**	26.0 ± 4.5	28.4 ± 6.7	0.3
**Sex (M/F)**	9/11	14/15	–
**Duration of diabetes (years)**	–	22.4 ± 13.9	–
**HbA**_**1C**_ **(mmol/mol)**	35.1 ± 2.8	57.3 ± 7.6	< 0.001***
**HbA**_**1C**_ **(%)**	5.4 ± 0.3	7.4 ± 0.7	
**Fasting blood glucose (mmol/L)**	4.6 ± 1.0	9.7 ± 3.7	< 0.001***
**Haemoglobin (g/dL)**	14. 3 ± 1.2	14.5 ± 1.2	1.0
**Triglycerides (mmol/L)**	1.4 ± 0.7	0.9 ± 0.4	0.145
**Alanine aminotransferases (IU/L)**	21.3 ± 6.1	22.8 ± 1.7	0.9
**eGFR (ml/min/1.73m**^**2**^**)**	78.09 ± 10.05	83.5 ± 9.8	0.259
**Systolic blood pressure (mmHg)**	117.5 ± 14.0	127.5 ± 9.4	0.005**
**Diastolic blood pressure (mmHg)**	74.8 ± 8.3	76.8 ± 8.8	0.3
**CD45**^**dim**^**CD34**^**+**^**CD133**^**+**^**/100 lymphocytes (cEPCs)**	0.09 ± 0.03	0.02 ± 0.01	< 0.001***
**Proangiogenic cells (PACs)**	44.9 ± 22.3	14.4 ± 7.6	< 0.001***
**Fibronectin adhesion assay per hpf**	68.6 ± 34.5	33.9 ± 14.7	0.017**

Values are presented as means ± SD; unpaired *t* test. T1DM, type 1 diabetes mellitus; HbA_1c_, glycated haemoglobin; CD, cluster of differentiation; cEPCs, circulating endothelial progenitor cells; hpf, high power field. *p < 0.05; **p < 0.01; ***p < 0.001

#### Cytokine characteristics of study participants

The levels of IL7 (2.3 ± 0.6 vs 1.4 ± 0.6; *p* = 0.005), IL8 (4.7 ± 1.3 vs 2.8 ± 0.5; *p* = 0.003), TNF-α (1.6 ± 0.2 vs 1.4 ± 0.2; *p* = 0.041) and VEGF-C (63.2 ± 20.3 vs 50.8 ± 48.2; *p* = 0.013) were detected to be significantly greater in T1DM patients compared to controls as reported previously by us [11]. The mRNA expression of *CXCR1* and *CXCR2* were 4.3 (*p* = 0.009) and 2.3 (*p* < 0.001) fold-change higher in T1DM patients, respectively which was previously documented by us [11].

#### Vascular health

T1DM patients had lower angiogenic markers: cEPC/CD45^dim^CD34^+^CD133^+^ cells (*p* < 0.001), PACs (*p* < 0.001) and FAA (*p* = 0.017) compared to healthy controls (Table 1). Missing cytokine data of one healthy control was addressed and calculated as the mean of healthy control group.

#### MiR-424-5p expression in T1DM

**Fig. 1:**
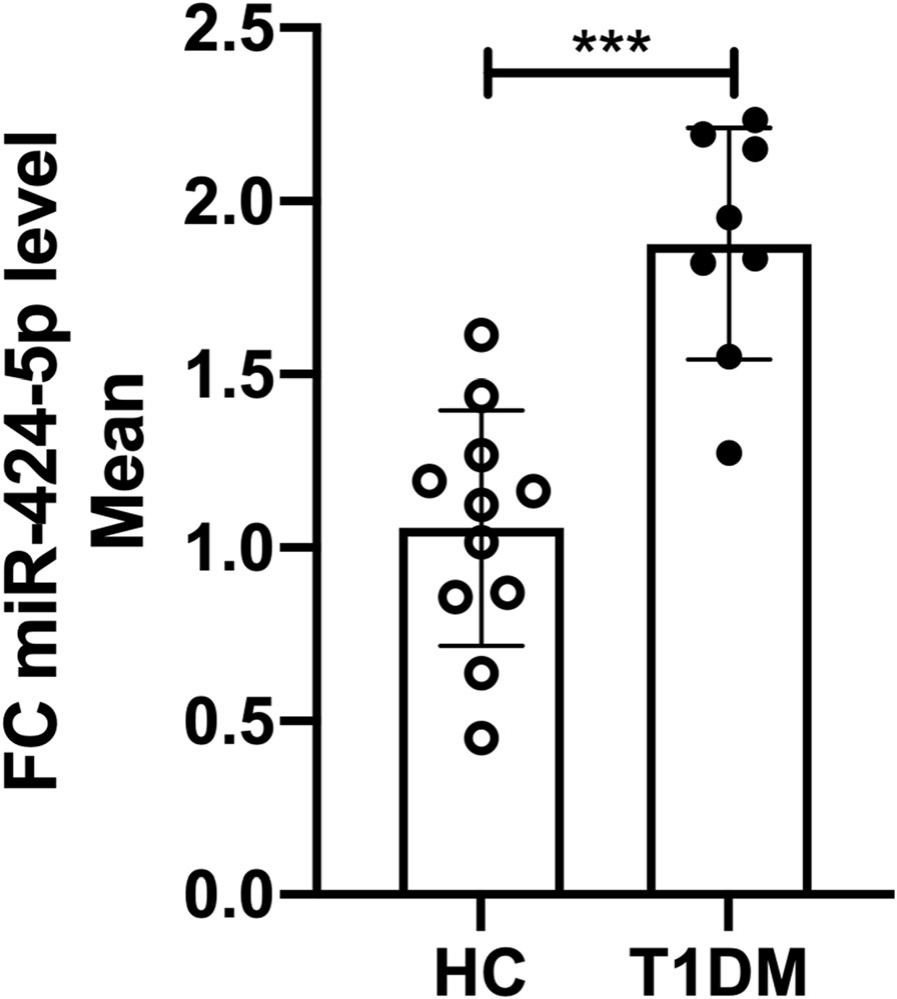
Comparison of miR-424-5p expression between HC and T1DM patients in peripheral blood mononuclear cells (PBMCs). Data are represented as mean ± SD and analysed by unpaired *t* tests. ***p < 0.001

**Fig. 2:**
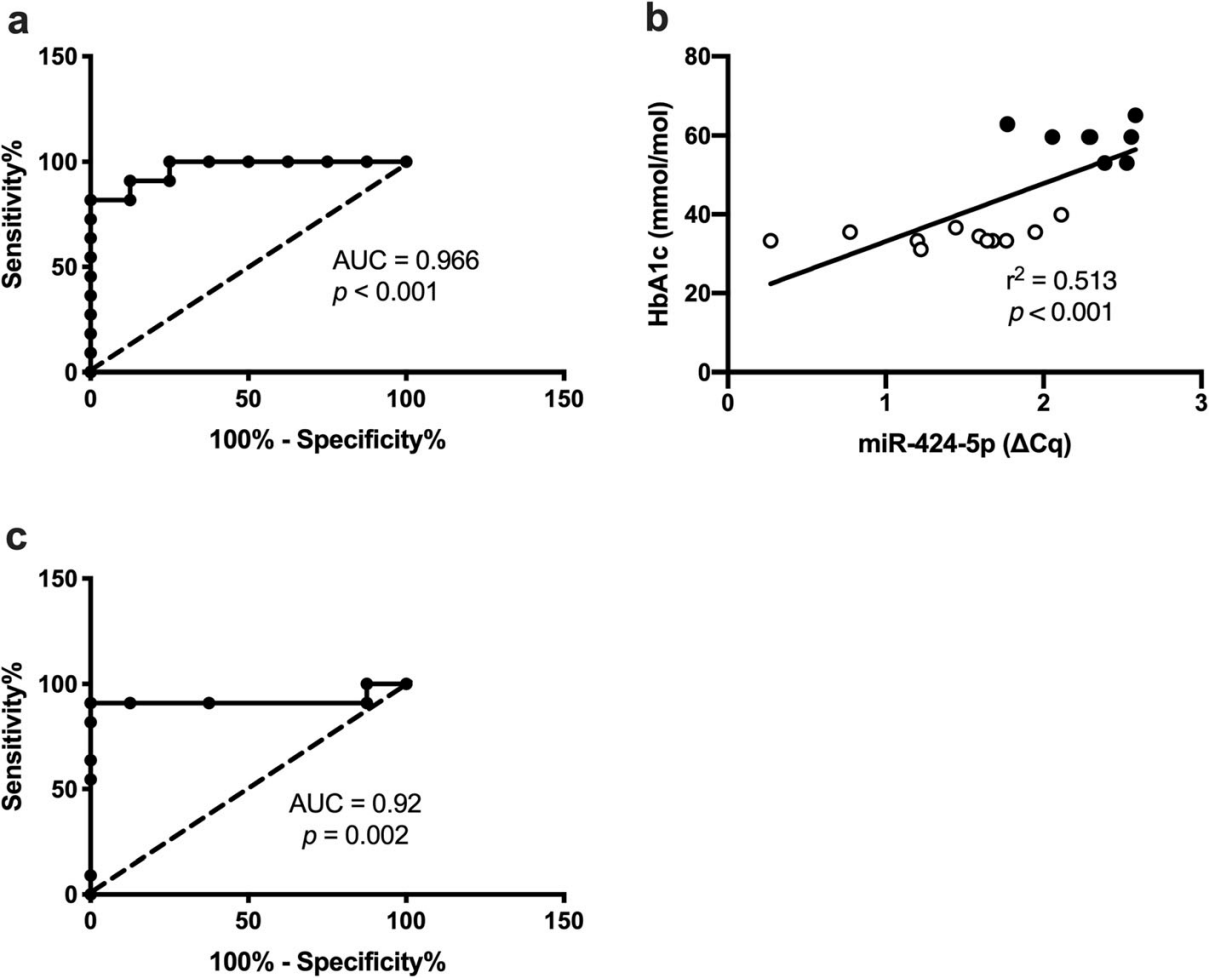
**a** ROC curve for miR-424-5p in both study groups. **b** Correlation between miR-424-5p in peripheral blood mononuclear cells (PBMCs) and HbA_1c_ (*r*^2^ = 0.599, *p* < 0.001) as a parameter to portray the glycaemic control across study groups, white dots indicated healthy control while black dots indicated T1DM. **c** ROC curve of HbA_1c_ to define upregulated miR-424-5p expression. Statistical analysis was performed using ROC curve analysis and linear regression analysis for correlation test. HbA_1c_, glycated haemoglobin

A significant upregulation of miR-424-5p was detected in the PBMCs of T1DM patients with 1.8-fold increase, *p* < 0.001 (Fig. 1), but not in plasma (data not shown). The correlation between miR-424-5p and systolic blood pressure was non-significant (*p* = 0.952). ROC curve analysis of miR-424-5p showed AUC = 0.966 (*p* < 0.001) with cutoff value of 1.49-fold, specificity = 87.5% and sensitivity = 90.9% (Fig. 2a) to predict T1DM. We found a positive correlation between miR-424-5p in PBMC and HbA_1c_: *r*^2^ = 0.513, *p* < 0.001 (Fig. 2b). Further ROC analysis of HbA_1c_ in upregulated and control miR-424-5p groups yielded AUC = 0.92 (*p* = 0.002) with HbA_1c_ cutoff value 46.5 mmol/mol (6.4%), specificity = 87.5% and sensitivity = 90.9% (Fig. 2c).

#### Correlations of miR-424-5p with proinflammatory cytokines and other parameters of vascular health

**Fig. 3:**
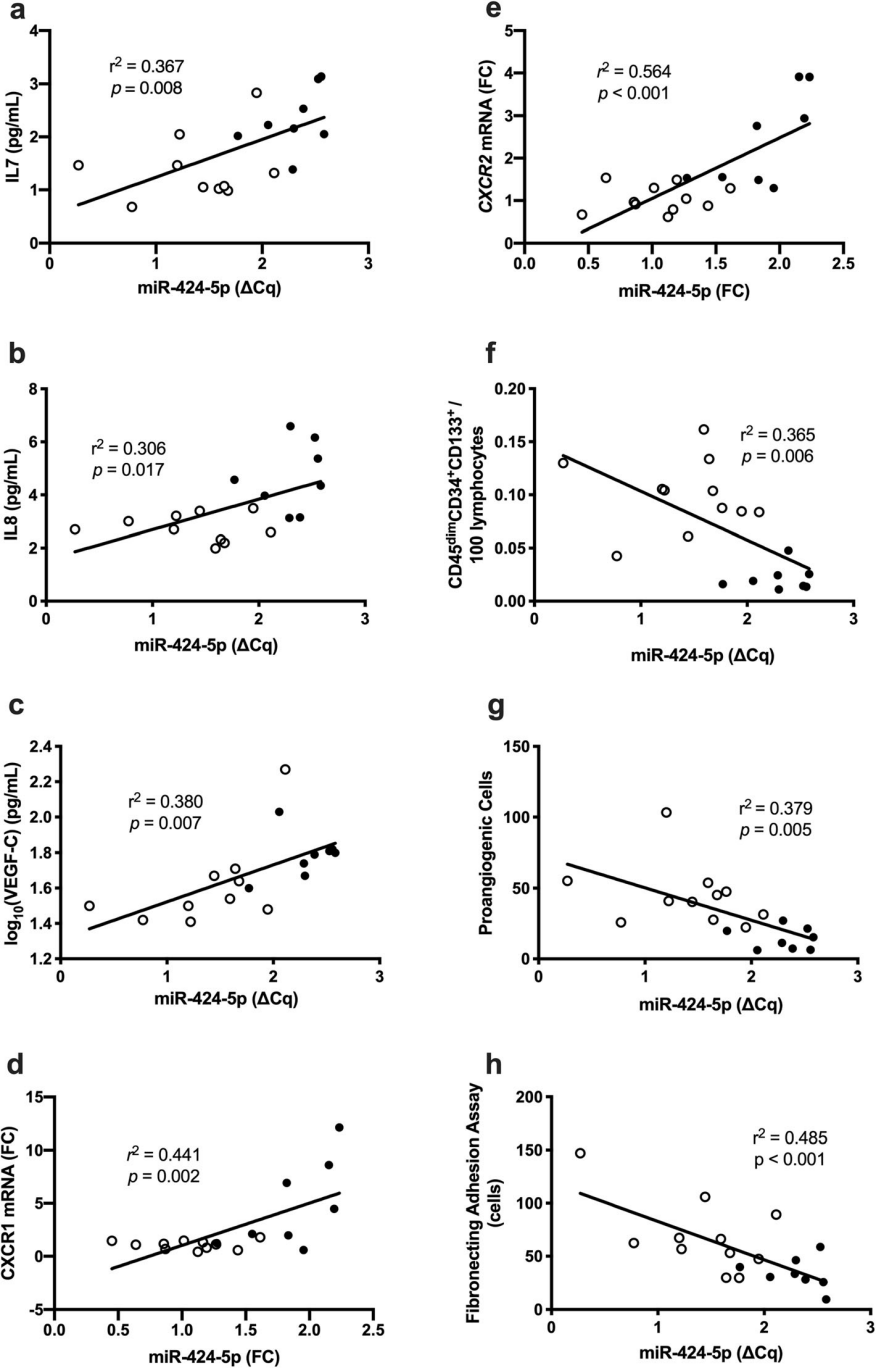
Correlation between miR-424-5p in peripheral blood mononuclear cells (PMBC and inflammatory cytokines **a** IL7 (*r*^2^ = 0.442, *p* = 0.003), **b** IL8 (*r*^2^ = 0.385, *p* = 0.006), **c** VEGF-C (*r*^2^ = 0.368, *p* = 0.008), **d** *CXCR1* mRNA in PBMC (*r*^2^ = 0.441, *p* = 0.002) and **e** PBMC *CXCR2* mRNA (*r*^2^ = 0.564, *p <* 0.001) and angiogenic markers: **f** CD45^dim^CD34^+^CD133^+^ (*r*^2^ = 0.43, *p* = 0.002), **g** proangiogenic cells (*r*^2^ = 0.418, *p* = 0.003), and **h** fibronectin adhesion assay (*r*^2^ = 0.396, *p* = 0.004), across study groups in the plasma. Statistical analysis was carried out using linear regression analysis. IL, interleukin; VEGF, vascular endothelial growth factor; CD, cluster of differentiation. White dots indicated healthy control while black dots indicated T1DM

Linear regression analysis of miR-424-5p in PBMCs showed positive correlation with IL7 (*r*^2^ = 0.367, *p* = 0.008) (Fig. 3a), IL8 (*r*^2^ = 0.306, *p* = 0.017) (Fig. 3b), VEGF-C (*r*^2^ = 0.380, *p* = 0.007) (Fig. 3c), *CXCR1* mRNA (*r*^2^ = 0.441, *p* = 0.002) (Fig. 3d) and *CXCR2* mRNA (*r*^2^ = 0.564, *p* < 0.001) (Fig. 3e) whilst negatively correlated with cEPCs (*r*^2^ = 0.365, *p* = 0.006) (Fig. 3f), PACs (*r*^2^ = 0.379, *p* = 0.005) (Fig. 3g) and their function in FAA (*r*^2^ = 0.485, *p* < 0.001) (Fig. 3h). Furthermore, no statistically significant association was seen with b-FGF, IFN-γ, IL-10, IL-16, IP-10, TIMP-1, TNF-α, E-selectin and P-selectin.

#### Functional pathway analysis by ingenuity pathway analysis

**Fig. 4:**
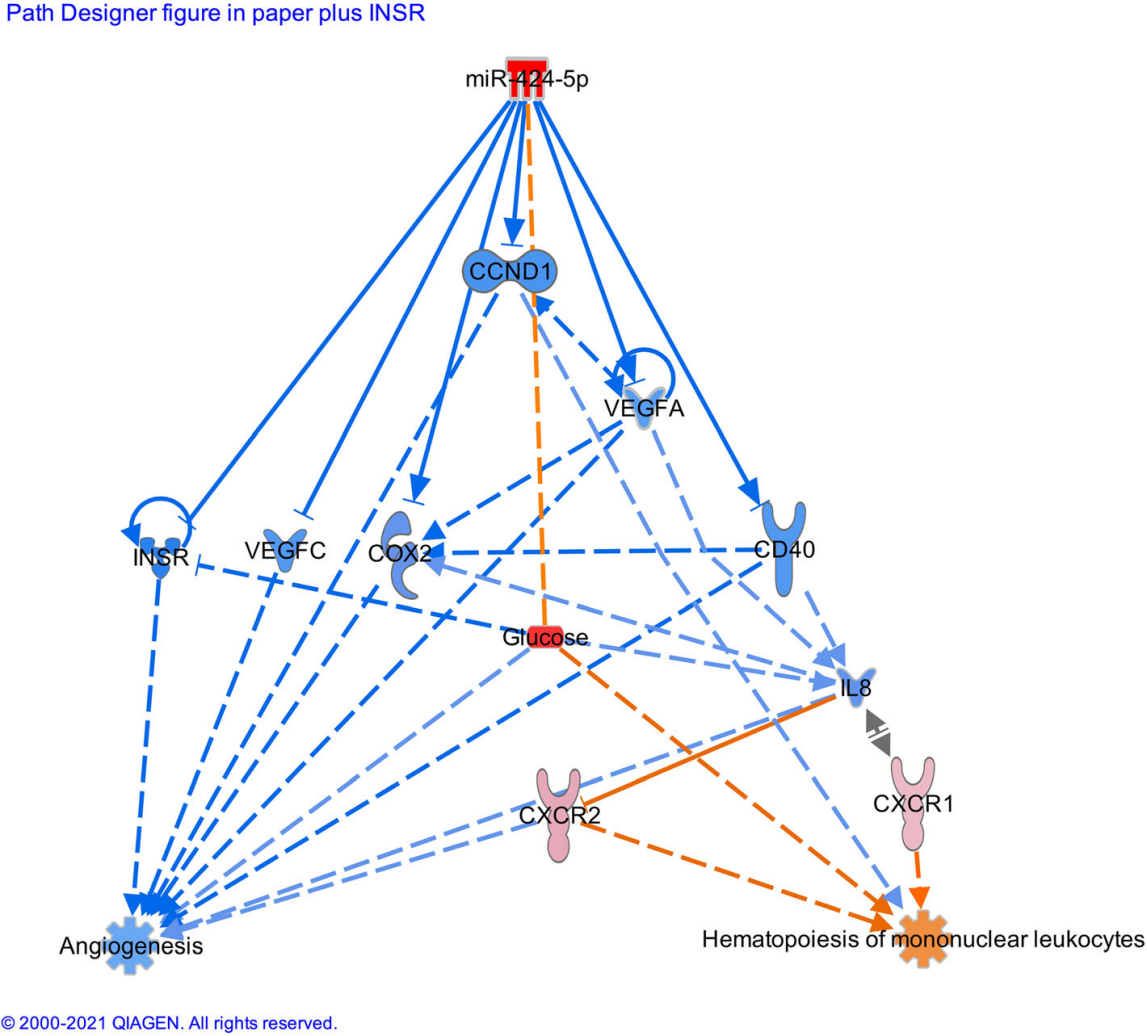
Ingenuity pathway analysis (IPA) prediction network of miR-424-5p using our data and its mRNA targets and cytokines supporting its involvement in cardiovascular disease. Three pathways were considered as relevant in this study: angiogenesis, haematopoiesis of monunuclear cells and insulin receptor signalling. CD, cluster of differentiation; COX2, cyclooxygenase-2; VEGF, vascular endothelial growth factor; CCND1, gene encoding cyclin D1; IL, interleukin, CXCR, chemokine receptors

Ingenuity pathway analysis (IPA) was used to perform the analysis of predicted mRNA targets based on miRNA sequence with or without additional supporting experimental evidence. Functional pathway analysis by IPA predicted miR-424-5p inhibiting angiogenesis and aiding mononuclear cells haematopoiesis. MiR-424-5p was strongly predicted to inhibit the mRNA expression of VEGF-A, which subsequently affected the expression of IL8, cyclooxygenase-2 (COX2) and gene encoding cyclin D1 which suppresses cell proliferation (CCND1). An inhibitory interaction with a weak evidence level was predicted between miR-424-5p and VEGF-C, inhibiting angiogenesis. The overexpression of miR-424-5p was predicted to supress the expression of CD40 mRNA, a crucial factor for antigen presenting cell, which works through IL8 to activate CXCR1 and CXCR2. CD40 was also speculated to activate COX2 to inhibit angiogenesis (Fig. 4).

## Discussion

In this study, well-controlled T1DM patients without CVD were considered as a model of subclinical CVD. Patients had diabetes sufficiently long enough to be suitable for studying early stages of CVD. Our data are in concordance with previous studies, showing an upregulation of proinflammatory cytokines IL7, IL8 and TNF-α along with the reduction of vascular health cEPCs, PACs and FAA in T1DM or diabetic animal models [7, 11, 25–28]. Others studied the upregulation of miR-424-5p in plasma in T1DM only and without further analysis [20]. We took our observation further and correlated our findings with indices of vascular health such as cEPCs (angiogenesis), fibronectin adhesion assay (cell adhesion) and proangiogenic cells (neovascularization potential). We are the first group to show that miR-424-5p was indeed upregulated in PBMCs of T1DM patients and this upregulation was associated negatively with indices of vascular health. Data was analysed as correlation between miR and other parameters.

### Confirmation of T1DM and CVD as inflammatory diseases

An upregulation of IL7, a lymphoid haematopoietic growth factor, recorded by us [11], has been shown to be associated with autoimmune diseases, such as multiple sclerosis and diabetes [29]. IL7 aided the expansion of autoreactive CD4 and CD8 T cells in the development of T1DM [29].

Moreover, significant elevations of serum TNF-α and IL8 in T1DM patients were observed in this study as described previously by us [11], confirming preceding research in T1DM [7, 25]. As a proinflammatory cytokine, TNF-α induced the secretion of IL8 to promote the inflammatory pathway [30]. IL8, in turn, recruited neutrophils and monocytes to the inflamed tissues by the activation of its receptors, CXCR1 and CXCR2 [31]. Supported by the upregulated mRNA of *CXCR1* and *CXCR2* in T1DM patients, this study suggests the presence of continuous induction of inflammatory signal, underlining the pathogenesis of most inflammatory diseases, such as T1DM and CVD [25, 32].

Patients included in our study had no evidence of CVD, but the presence of elevated pro-inflammatory cytokines is in line with the predisposition to asymptomatic CVD in these patients. Hence, our findings further confirm the inflammatory nature of T1DM and its contribution to increase the risk of developing CVD.

### MiR-424-5p association with vascular health

T1DM has been closely related with macrovascular complications such as myocardial infarction and defective vascular capacity, including delayed wound healing [3, 4]. The reduction in vascular health of T1DM patients, revealed by the diminished cEPCs, PACs and FAA, was previously reported by us [5]. Circulating EPCs, fraction of PBMCs, are cells which play an important role in maintaining endothelial integrity, aiding neovascularisation and regeneration of endothelial cells and predicting future CVD events [9, 33]. The inverse correlation with cEPCs may confirm animal experiments that miR-424-5p is antiangiogenic. Further evidence of antiangiogenic effect of miR-424 may come from the inverse correlation with in vitro vascular function such FAA or PACs demonstrating adhesion or neovascularization properties of cells involved in vascular health.

The *r*^2^ value varied for different linear regression models (Fig. 2b and Fig. 3) from 0.5 to 0.3. The possible explanation is that, as the number of data points was the same in all linear regression models, moderate *r*^2^ may be a reflection that less variation of a dependent variable is explained by the independent variable.

The studies proving causal relationship of miRs in vascular health in patients are still beyond our reach as mechanist experiments in patients are still not possible. However, both circulating EPCs and attached PACs originate from PBMCs; thus, it may well be possible that miR-424-5p are also overexpressed in those cells. Further studies are thus required to answer those important questions. The correlations found by us may confirm indirectly the results from animals’ research, in which miR-424 overexpression caused a significant decrease in proliferation of pulmonary artery endothelial cells [17]*.* We have shown inverse relation of miR-424-5p with cEPCs, PACs and FAA. In the hindlimb ischemia model, PACs cultured from BPBMCs not only secreted several cytokines such as VEGF and IL-8 (miR-424-5p gene targets (Fig. 4) involved in vascular health but also induced well-organised perfused vessels when transplanted which showed neovascularization activity [21].

Hence, considering the nature of miR-424-5p which is deleterious to vascular health when being overexpressed [18, 19], the upregulation of miR-424-5p in T1DM patients may contribute to the increased risk of CVD in T1DM. Recent studies in unstable atherosclerotic plaque from patients with peripheral arterial disease identified highly expressed Notch ligand Delta-like 4 (Dll4) and miR-424-5p, complex associated with disease progression [16].

### MiR-424-5p and cytokines

Our results showed direct relation between miR-424-5p in PBMCs and concentration of circulating IL-8, IL-7 and VEGF-C cytokines. The relationship between miR-424-5p expression and cytokines has been demonstrated in previous studies.

In cancer, dysregulation of suppressor of cytokines signalling (SOCS) protein expression was common. It was reported that miR-424-5p expression was essential in mediating IL-8/STAT5/SOCS2 pathway in inducing cell migration and cellular invasiveness in oral squamous cell carcinoma. IL-8 displayed its function by inducing the expression of miR-424-5p and hence modulating SOCS2/STAT5 signalling [34]. Hence, the upregulation of miR-424-5p in T1DM may be caused by the chronic IL-8 elevation. However, this requires confirmation in non-cancerous tissues.

We found a positive linear relation between miR-424-5p and mRNA *CXCR1* and mRNA *CXCR2* and between miR-424-5p and IL-8 cytokine. Moreover, the activation of CXCR1/2 chemokine receptors by IL8, expressed in granulocytes, monocytes and microphages led to trafficking cells to inflammatory sites including arteriosclerotic plaques [35, 36]. Our group is first to report the correlation of miR-424-5p with mRNA *CXCR1* and *CXCR2* in PBMCs whilst circulating IL-8 was also elevated. This finding may point toward atherogenic action of elevated miR-424-5p, mRNA *CXCR1* and *CXCR2*, especially as IL-8 cytokine has been found to be mitogenic and chemotactic for smooth muscle cells [37].

We have shown a direct linear relationship between miR-424-5p and IL-7 concentrations. The elevated levels of IL-7 have been shown in patients with acute coronary syndrome. Furthermore, IL-7 enhanced the expression of inflammatory chemokines in monocytes and PBMCs in these patients [38]. Others suggested IL-7 to have predictive value for mortality from cardiogenic shock following myocardial infarction [39]. Cytokine IL-7 was involved in the inflammatory pathway consisting of monocyte trafficking into atherosclerotic plaques [40]. In addition, in laboratory experiments, IL-7 increased cell adhesion molecules and monocyte chemoattractant in endothelial cells and promoted monocyte adhesion and trafficking of macrophages into aortas of ApoE^−/−^mice whilst IL-7 receptor blockade reversed that process [41]. Integrative analysis of miRNA-mRNA network in high-attitude retinopathy predicted IL-7 receptor to be a target for both miR-369-3p and miR-424-5p [42]. However, retinopathy is a unique model and reflects small vessel disease; thus, further studies are necessary on IL-7R and miR-424-5p in large vessel disease (CVD).

### PBMC miR-424-5p expression in T1DM patient

The strength of our study lies in studying in parallel the expression of miR-424-5p in both PBMC and plasma. We have shown for the first time that the upregulation of miR-424-5p in PBMC, but not in plasma, of T1DM patients (Fig. 1).

Although the upregulation of serum miR-424-5p in DM patients has been recorded in previous research [20], it is crucial to ensure the purity of cell-free-plasma before analysing the plasma samples using RT-PCR, which we have employed in this study. This is particularly important since miRNA analysis in platelet-free-plasma and standard plasma was reported to yield different results [43]. Thus, discrepancy between above-mentioned studies may be related to pre-analytical methodology used.

### MiR-424-5p and T1DM

We are the first group to report in T1DM patients raised miR-424-5p in PBMCs. Our results are supported by the evidence from recently published study in rodent model of T1DM. Specifically, in Wistar male rats induced by streptozotocin (model of T1DM), hsa-miR-424-5p mimic plasmid and hsa-mir-424-5p inhibitor plasmid were designed and injected. The results of that study showed that hsa-miR-424-5p mimic group had the highest expression of hsa-miR-424-5p in lymphocytes and hsa-miR-424-5p linked to programmed cell death 1 signalling (PD-1), which stimulated the immune response through the mechanistic target of rapamycin kinase signalling pathway (mTORC) and therefore participated in the pathogenesis of T1DM [44].

Furthermore, another study reported the upregulated miR-424-5p as a biomarker for positive autoantibody status, in relatives of T1DM patients, who subsequently developed type 1 diabetes during the follow-up [20]. Recent study showed higher urinary exosomal miR-424 expression in T1DM in children than in healthy controls [45]. Our study assessed the ROC curve of miR-424-5p in T1DM and healthy controls. We obtained an AUC of 0.966 (*p <* 0.001), and data showed that miR-424-5p is a strong biomarker for T1DM with 90.9% sensitivity and 87.5% specificity (Fig. 2a). This is consistent with a positive association between glycaemic control, miR-424-5p level and prediction on IPA pathway analysis. The most important proof of our finding are the intervention studies, in which the administration of high glucose concentration in either human mesenchymal stem cells or mouse models, led to miR-424-5p rise, confirming that miR-424-5p is caused by hyperglycaemic state [46, 47].

Although our analysis points toward miR-424-5p being a biomarker for T1DM, there may be an alternative conclusion to our finding as one has other means of diagnosing T1DM including HbA1c. We postulated that miR-424-5p is a biomarker for early subclinical CVD and the difference in miR-424-5p expression could be attributed to the presence or absence of hyperglycaemia.

Consequently, we tested the above-mentioned hypothesis by assessing the cutoff value of HbA_1c_, for which miR-424-5p was upregulated to determine if miR-424-5p could itself predict CVD in our cohort of subjects. It is of interest that the value of HbA_1c_ 46.5 mmol/mol (6.4%) achieved from ROC curve analysis also signified the turning point for development of diabetes and commonly phrased as prediabetes state [48]. To validate our original finding, the values of HbA_1c_ used to define the diabetes state are equally defined by the development of microvascular complication [48, 49]. In our study of well-characterised controls and diabetic patients, we found independently by using miR-424-5p, the biochemical set point for diabetic complications. This cutoff defined an increased CVD risk and appeared to coincide within the pre-diabetes range (42–47 mmol/mol or 6.0–6.49%). Despite the small study size, this independent analysis can be used to confirm that miR-424-5p is a biomarker for the beginning of CVD. Others also reported miR-424-5p to be a biological marker of intracranial aneurism [50] or predictor of risk of fatal myocardial infarction whilst combined with Framingham score [51].

Thus, this finding has practical application, as reliable diagnosis of subclinical CVD is notoriously difficult without using invasive methods. Therefore, miR-424-5p can act as either biomarker of CVD onset or CVD progression or both. The upregulation of miR-424-5p emphasises the increasing cardiovascular risk with the presence of T1DM even in the absence of clinical CVD.

### Prediction model: functional pathway analysis of miR-424-5p in relation to cardiovascular function

IPA predicted miR-424-5p to regulate angiogenesis and haematopoiesis of mononuclear leukocytes (Fig. 4). The upregulation of miR-424-5p was predicted to inhibit mRNA expression of CD40, VEGF-A, VEGF-C and COX2. These interactions result in an activation of CXCR1 and CXCR2 along with subsequent protein inhibitions (cyclin D1, IL8) to halt angiogenesis and activate mononuclear leukopoiesis. This prediction is in line with our mRNA analysis, which showed an upregulation of *CXCR1* and *CXCR2* mRNA concentrations following an upregulation of miR-424-5p in T1DM patients (Fig. 3d, e).

The predicted interaction of upregulated miR-424-5p to downregulate angiogenic growth factor, VEGF-C, was based on incomplete matching sequence (weak evidence). This is also in line with the previous non-diabetic mice study, showing the upregulated miR-424 to reduce VEGF expression in endothelial cells [52]. However, this prediction does not take into account the influence of diabetes on VEGF regulation.

Therefore, it is not surprising that this part of the network prediction contradicts our data which shows an upregulation in VEGF-C and no significant change in of VEGF-A and VEGF-D that we have studied (data not shown). Furthermore, contrary to the IPA prediction, our findings are also supported by a positive correlation between miR-424-5p and VEGF-C concentration. Moreover, if endothelial growth factors such as VEGF-A, VEGF-C and VEGF-D would be upregulated, this would have led to an elevation in cEPCs and PACs, which was not the case in our participants. Hence, we speculate that the elevation in VEGF-C is more likely to be compensatory mechanism in T1DM patients to induce angiogenesis and it is regulated by different mechanism, which we have not studied here.

**Fig. 5:**
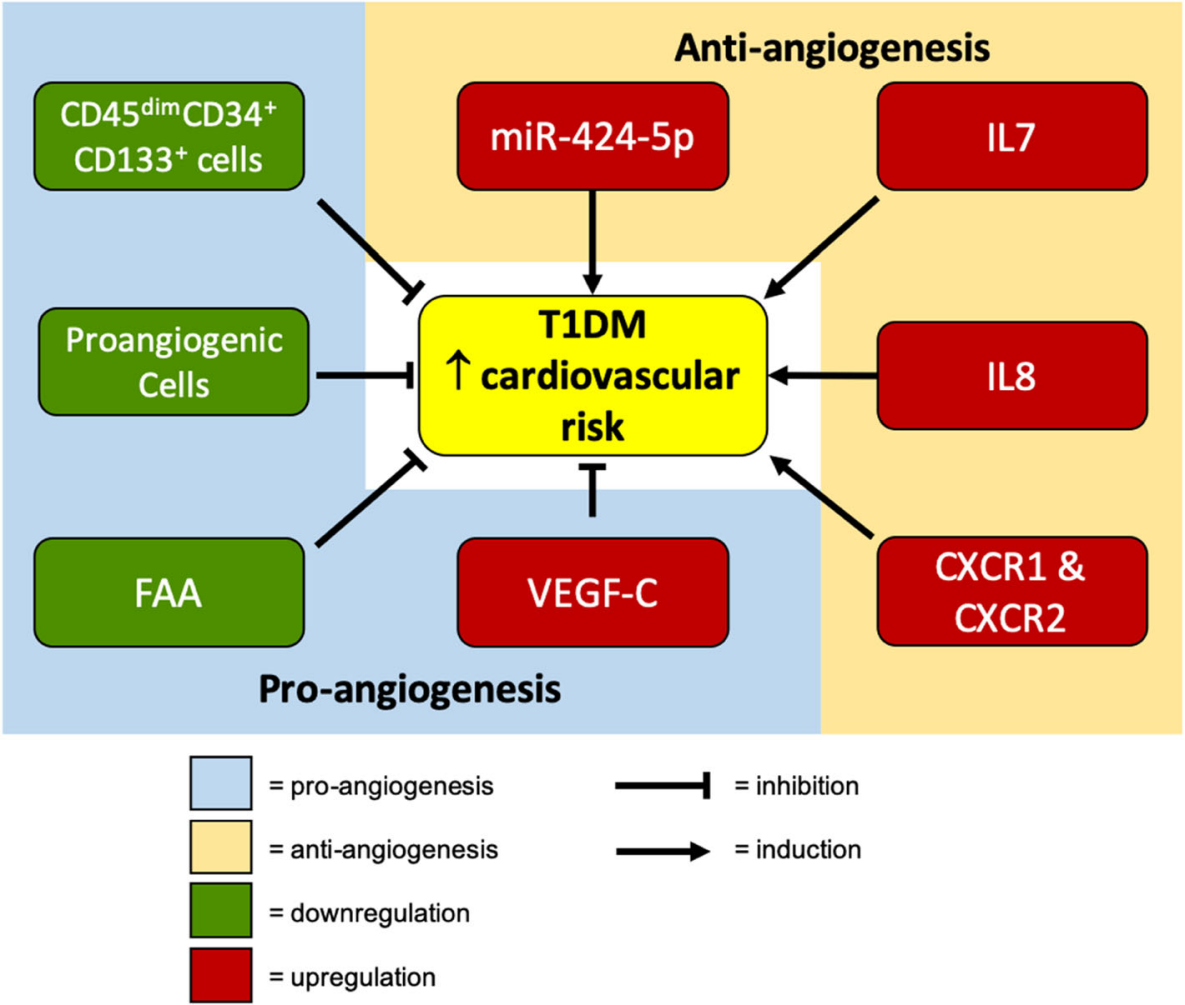
Summary of our study results in T1DM. The upregulated miR-424-5p, cytokines (VEGF, vascular endothelial growth factor; IL, interleukin) or their receptors (CXCR, chemokine receptor) contributed to increased cardiovascular risks in T1DM patients whilst vascular health—fibronectin adhesion assay(FAA), proangiogenic cells and CD45^dim^ CD34^+^ 133^+^cells—was reduced in the same patients with T1DM, type 1 diabetes mellitus, thus displaying impaired vascular repair

Our interpretation of VEGFs findings is in line with the previous research showing VEGFs elevation in diabetes [27, 28] and supported by our own in vitro study [26]. In diabetes, the impairment in angiogenesis, despite of elevated VEGFs, is due to the downregulation of VEGF receptors (VEGFR) and impairment in VEGF-VEGFR signalling. This leads to upregulation of the upstream signal, VEGFs, as shown by others [26–28]. The summary of our research is graphically represented in Fig. 5.

The limitation of this study includes the use of cross-sectional data. Hence, we recommend a future longitudinal study to investigate the role of miR-424-5p in progression of CVD risk in T1DM patients.

### Contribution/causation

MiR-424-5p has effect on 32 pathways (mirPath-v3) of which many are interrelated. Since our clinical study relies on comparisons and correlations, the contribution or causality may be drawn from miR-424-5p target genes binding site as listed in Table 2. MiR-424-5p has binding sites in 5 target genes of interest. The highest number of binding sites was found among insulin receptor signalling pathway. Thus, it appears that miR-424-5p has causal role in glucose metabolism via insulin receptor pathway. The next causal effect of miR-424-5p is on CCND1 gene, which belongs to the highly conserved cyclin family involved in smooth muscle proliferation (VSMCs). The further causal effect is via CD40, gene coding for the TNF-receptor superfamily. This receptor has been found to be essential in mediating a wide variety of immune and inflammatory responses. The other causal role of miR-424-5p is on an important target gene (COX2), precursor for prostacyclin synthesis, which is involved in inflammation. Last but not the least was the causal effect of miR-424-5p in our model on VEGFA, inherently involved in angiogenesis [53].

**Table 2:** Predicted consequential pairing of target region in the transcript and miR-424-5p

Target gene	Representative transcript	Gene name	Transcript position	Predicted consequential pairing of target region Transcript (top) and miRNA (bottom)	Site type
CCND1	ENST00000227507.2	Cyclin D1	1961–1967 3′UTR	(Transcript) 5′ CCAUUUUCUUAUUGC**GCUGCUA**C 3′ (miRNA) 3′ AAGUUUUGUACUUAA**CGACGA**C 5′	7mer-A1
			2033–2040 3′UTR	(Transcript) 5′ CUCUUUC**ACAU**UGUU**UGCUGCU**A 3′ (miRNA) 3′ AAGUUU**UGUA**CUUA**ACGACGA**C 5′	8mer
VEGFA	ENST00000372067.3	Vascular endothelial growth factor A	276–283 3′UTR	(Transcript) 5′CCAUUUUAUUUUUCU**UGCUGCUA** 3′ (miRNA) 3′ AAGUUUUGUACUUA**ACGACGA**C 5′	8mer
CD40	ENSG00000101017	CD40, TNF receptor superfamily member 5	61–71 3′UTR	(Transcript) 5′ CUUCCUGGCUCC**AACA**C**UGCUGCU**C 3′ (miRNA) 3′ AAGUU**UUGU**ACUUA**ACGACGA**C 5′	7mer
COX2	ENSG00000073756 (PTGS2)	Cyclooxygenase-2	165–179 3′UTR	(Transcript) 5′ UGAUUUGUUAUU**AACA**U**UGA**UC**GCUG** 3′ (miRNA) 3′ AAGUU**UUGUACU**UA**ACGAC**GAC 5′	6mer
INSR	ENSG00000171105	Insulin receptor	4218–4241 3′UTR	Transcript) 5′UCC**UCAAA**U**UGA**CCA**AU**AGC**UGCUGC** 3′ (miRNA) 3′ A**AGUUU**UGU**ACUUAACGACGA**C 5′	7mer
			3844–3871 3′UTR	(Transcript) 5′**UCA**U**CAUGA**UCACUGAUUAC**UUGCUGCU**C3′ (miRNA) 3′ A**AGU**UUU**GUACU**U**AACGACGA**C 5′	8mer

TargetScan Human, release 7.1 (www.targetscan.org) and Diana-TarBase v8 (http://carolina.imis.athena-innovation.gr/diana_tools/web/index.php?r=tarbasev8%2Findex) databases were used to predict the interaction sites between the transcripts and miR-424-5p

Although reporting correlations from patients does not provide definitive answers to the mechanism involved, we believe it is pivotal, in exploring new research avenues, as it is generated by clinical findings and therefore may shorten the time from the bench to bedside.

## Conclusions

Taken together, this study in patients with T1DM validated animal research. Our data indicates that miR-424-5p may be used as a sensitive biomarker for subclinical cardiovascular disease and has potential as a therapeutic or monitoring target in CVD. The study conclusions are drawn from correlations found and supported regarding contribution/causation by binding sites for miR-424-5p in insulin receptor pathways, CD40, CCND1, VEGFA and COX2 genes.

## Data Availability

The datasets used and/or analysed during the current study are available from the corresponding author on reasonable request.

## Abbreviations

CCND1: Gene encoding cyclin D1
CD: Cluster of differentiation
cEPC: Circulating endothelial progenitor cells
COX2: Cyclooxygenase-2
CVD: Cardiovascular disease
CXXR: Chemokine receptor
FAA: Fibronectin adhesion assay
HbA_1c_: Glycated haemoglobin
IL: Interleukin
IPA: Ingenuity pathway analysis
miR: microRNA
PAC: Proangiogenic cell
PBMC: Peripheral blood mononuclear cell
ROC: Receiver operating characteristic
T1DM: Type 1 diabetes mellitus
VEGF: Vascular endothelial growth factor
VEGFR: VEGF receptors
